# Five-year follow-up of a randomized weight loss trial on a digital health behaviour change support system

**DOI:** 10.1038/s41366-025-01742-4

**Published:** 2025-03-15

**Authors:** Eero Turkkila, Taru Pekkala, Heta Merikallio, Marko Merikukka, Laura Heikkilä, Janne Hukkanen, Harri Oinas-Kukkonen, Tuire Salonurmi, Anna-Maria Teeriniemi, Terhi Jokelainen, Markku J. Savolainen

**Affiliations:** 1 Research Unit of Biomedicine and Internal Medicine, University of Oulu, Oulu, Finland; 2 Medical Research Center Oulu, University of Oulu and Oulu University Hospital, Oulu, Finland; 3 Research Service Unit, Oulu University Hospital, Oulu, Finland; 4 Department of Sports and Exercise Medicine, Oulu Deaconess Institute Foundation sr, Oulu, Finland; 5 Research Unit of Population Health, University of Oulu, Oulu, Finland; 6 Oulu Advanced Research on Service and Information Systems, University of Oulu, Oulu, Finland; 7 Research Unit of Health Sciences and Technology, University of Oulu, Oulu, Finland; 8 Psychiatry, University of Oulu and Oulu University Hospital, Oulu, Finland

**Keywords:** Lifestyle modification, Clinical trials

## Abstract

**Background:**

The weight regain after successful weight loss is a common challenge. This study aimed to assess the long-term effectiveness of a web-based health behaviour change support system (HBCSS) utilizing persuasive systems design (PSD) and methods of cognitive behavioural therapy (CBT). We have previously demonstrated the two-year effectiveness of the HBCSS.

**Methods:**

In total, 532 participants with overweight or obesity (BMI 27–35 kg m^−2^) were split into three groups with different intensities of intervention: CBT-based group counselling, self-help guidance (SHG), and usual care. These groups were further divided into HBCSS and non-HBCSS groups. The HBCSS was a 52-week programme. The follow-up took five years in total.

**Results:**

Mean weight change (%) (95% CI) from baseline among HBCSS and non-HBCSS users was 1.5 (−0.02 to 2.9), *p* = 0.056 and 1.9 (0.3–3.3), *p* = 0.005, respectively, at five years. Of the six groups, the SHG group without HBCSS had a statistically significant increase in weight (%) from baseline at five years (3.1, 95% CI 0.6 to 5.6, *p* = 0.010). The other groups did not have a significant increase in weight. There was no significant difference between groups at five years in weight. Fewer blood pressure medications were started over the five-year period in HBCSS group (*p* = 0.046).

**Conclusion:**

The 12-month HBCSS intervention was not able to maintain reduced weight better than non-HBCSS at 5 years. However, there were significant weight difference favouring HBCSS over the whole 5-year period. The decrease in the need for antihypertensives suggests that the significant weight loss by HBCSS at early years has a health-promoting legacy effect.

## Introduction

The challenge in treating overweight and obesity is to sustain reduced weight. Obesity should be approached as a chronic condition requiring longer-term support and treatment [1]. Lifestyle interventions are effective in achieving weight loss in the short term, but preserving the results is often challenging [2–4]. Better weight management methods are needed to ensure long-term weight maintenance. Currently, there is no universally effective approach to weight maintenance [4].

Digital technologies have significant potential for engaging people with health behaviour change and helping weight reduction [1, 5]. Mobile application is an easily accessible and potential platform for obesity treatment as most adults own a smartphone and half have downloaded a health-related mobile app [5, 6]. There is evidence that web-based interventions may be useful in weight loss and maintenance in the short term [1, 3]. A meta-analysis showed that digital interventions may be more effective in weight loss than a non-digital intervention in the short-term [7]. Some studies have shown that interactive technology interventions help reduce weight and maintain weight loss for up to two years [3, 8]. Currently, personal contact intervention, including telephone and face-to-face contact, seems to be the most effective both in weight loss and maintenance [3, 9].

We previously performed a randomized clinical trial on the effectiveness of a web-based health behaviour change support system (HBCSS) that utilizes persuasive systems design (PSD) and methods of cognitive behavioural therapy (CBT). We had six intervention groups: CBT-based group counselling, self-help guidance-based group counselling (SHG), and usual care further divided into the HBCSS and non-HBCSS groups [8]. The trial demonstrated that one-year treatment with HBCSS was effective for weight loss, and remarkably, the weight loss result was maintained for one year after the intervention, even when HBCSS was utilized without any face-to-face counselling [8]. Weight reduction in CBT-based counselling with HBCSS was 4.1% at 12 months and 3.4% at 24 months. HBCSS alone achieved a 1.6% weight reduction at 24 months.

Here we report the five-year outcome of the HBCSS and/or group counselling treatment. Our primary aim was to assess the long-term effectiveness of HBCSS in weight control.

## Materials and methods

### Participants and randomization

**Fig. 1:**
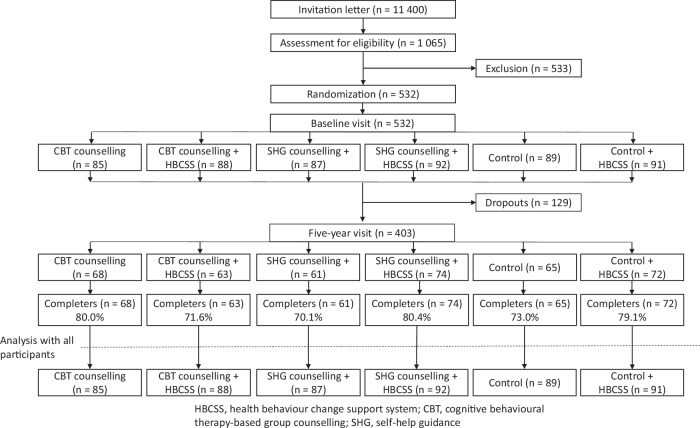
Participant flow during the 5-year follow-up. The flowchart represents all groups, either with or without HBCSS. The completers are the participants measured at baseline and the 5-year visit. Detailed changes in participants, including the 1- and 2-year visits, are presented in a previous publication [8].

An invitation letter was sent to 11,400 people aged 20–60 living in Oulu, Finland, and 1065 volunteers were assessed for eligibility. The inclusion criteria were BMI (27–35 kg m^−2^), access to the internet, no health-related restrictions to losing weight, and no other treatment for obesity. After exclusions due to significant illness or abnormal laboratory values, 532 participants were randomized into six groups in two phases (Fig. 1). First, participants were divided into three groups, one control group and two groups with different face-to-face group counselling methods. Secondly, these three groups were divided into web-based HBCSS users and non-users.

At baseline, 532 participants were randomly assigned to the study arms. Between the baseline and five-year visits, 129 participants (24.2%) dropped out. The completers are the participants measured at baseline and the 5-year visit. All randomized participants (except for one) were approached and invited to the five-year visit, even if they had not visited the research centre after the baseline visit.

### Intervention and treatment arms

CBT group counselling included eight sessions with qualified or student clinical nutritionists who had knowledge of CBT. Treatment aims were to (1) lose and maintain 5% weight loss, (2) improve eating behaviour, and (3) decrease health risks related to overweight and obesity (e.g., diabetes, cardiovascular diseases). Group counselling with SHG included two sessions with a registered nurse. The sessions were based on Elvira counselling model, which utilizes constructive learning theory and stages of change model [10, 11]. The sessions aimed to help with lifestyle changes and eating behaviour. The control group received usual care usually given in primary health care, including information about health and weight management and metabolic syndrome (a leaflet and personal laboratory results).

The web-based HBCSS was a 52-week programme based on PSD combined with CBT methods. The programme guided the participants towards a better lifestyle, weight loss, and a healthier state. The system included motivative functions, e.g., the possibility to set goals and follow progress. It also provided information about health and healthy lifestyle-related topics. Software features implemented in the system supported users to carry out their primary task, computer-human dialogue, perceived credibility, and social influence. These included specifically design principles of self-monitoring, reduction, tunnelling, tailoring, praise, reminders, suggestion, liking, verifiability, social learning, and social facilitation [12, 13]. Detailed information about HBCSS, treatment arms and sample size calculations are presented in the previous publication [8].

### Measurements

Participants in each group were followed up at baseline and one year, two years, and five years after the baseline visit in the research centre at the Oulu University Hospital. Waist circumference, weight, and blood pressure were measured at each visit. Height was measured at baseline and five years. Information about the medications of the participants was collected at each visit.

### Statistical methods

The primary outcome was the weight loss (kg) from the baseline to five years. Changes in weight from baseline to five years were compared in six groups among all participants based on the intention-to-treat principle.

A linear mixed-effect model (LMM) (as primary analysis), where (six) group and time were fixed effect factors was used to examine the differences in weight from baseline to 5 years adjusting for age and sex [14]. Group and time interaction was included in the model to examine whether mean change over time was different between groups. Data included some missing values, but they were assumed to be at random. The same model was used with imputed values (both the Last Observation Carried Forward (LOCF) and Return-To-Baseline (RTB)) where data were missing and for completers as sensitivity analyses. Primary analysis and sensitivity analyses were based on intention-to-treat principles. Primary analysis was also used to examine the differences between HBCSS groups and non-HBCSS groups.

Furthermore, the supplementary net incremental Area-Under-the-curve (AUC) analysis was used to compare weight changes during the five years of follow-up in HBCSS and non-HBCSS groups. The AUC analysis visualises and outlines the weight change trend over the five-year period and was therefore performed as an off-protocol analysis. The calculations of incremental AUCs subtracted the baseline weight from the weight at one-year, two-year, and five-year time points. If the subtraction of the baseline from the subsequent time point resulted in a negative value, leading to a negative peak in incremental AUC calculation, the resulting negative peak area was subtracted from the positive peak area (net area calculation). The inclusion criteria for AUC analysis were that the baseline and at least one other time point measurement were available. The five-year measure got a value of 0 (no change in weight) if missing. The net areas from AUC analysis were run by analysis of variance (ANOVA). Intragroup changes were analysed with One-Sample *T* Test. IBM SPSS Statics version 26.0 (IBM Corp., Armonk, NY, USA) was used for the analyses. GraphPad Prism 9 (GraphPad Software Inc., San Diego, CA, USA) was utilized for the AUC analyses.

In addition, differences in the weight change proportions between study arms were calculated with Chi-square analysis. The changes in the number of lipid, cardiovascular and diabetes medications between HBCSS and non-HBCSS groups were analysed with the Chi-Square Test. *p-*values less than 0.05 were considered statistically significant.

## Results

### Baseline characteristics

The mean age (with standard deviation) of study subjects (*n* = 532) was 45.9 ( ± 9.9) years, mean body weight was 89.5 (±11.2) kg, and mean BMI was 30.4 ( ± 2.1) kg m^−2^. Of all participants, 50.8% (*n* = 270) were men, and 49.2% (*n* = 262) women. At baseline, 52.3% of the participants were living with obesity (BMI > 30 kg m^−2^) and 47.7% were living with overweight (BMI 27–30 kg m^−2^). Detailed information about baseline characteristics is presented in the previous publication [8].

### Primary outcome

**Fig. 2:**
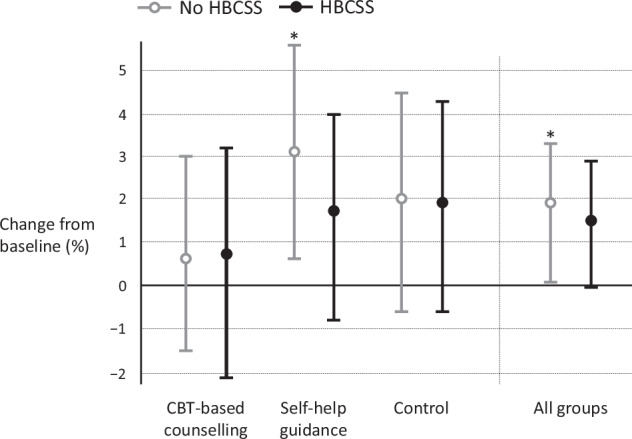
Effect of digital lifestyle counselling on weight—5-year outcomes of a randomized clinical trial. Linear mixed-effect model was used to examine the differences in weight change (kg). Data is presented in means (%) with 95% confidence intervals. Grey line, No HBCSS; black line, HBCSS. HBCSS, health behaviour change support system. *p < 0.05.

Among all participants (*n* = 532) included in the analysis, there was no significant difference between the six groups at five years (*p* > 0.05) in weight change (Fig. 2, Supplementary Table 1). The SHG group without HBCSS had a statistically significant increase in weight (%) from baseline (3.1, 95% CI 0.6–5.6, *p* = 0.010). The other groups did not have a significant increase in weight. There was no statistically significant difference between HBCSS group and non-HBCSS group. Mean weight change (%) (95% CI) from baseline among HBCSS and non-HBCSS users was 1.5 (−0.02 to 2.9), *p* = 0.056 and 1.9 (0.3 to 3.3), *p* = 0.005, respectively, at five years. Results of sensitivity analyses were similar. Those results are not shown.

**Fig. 3:**
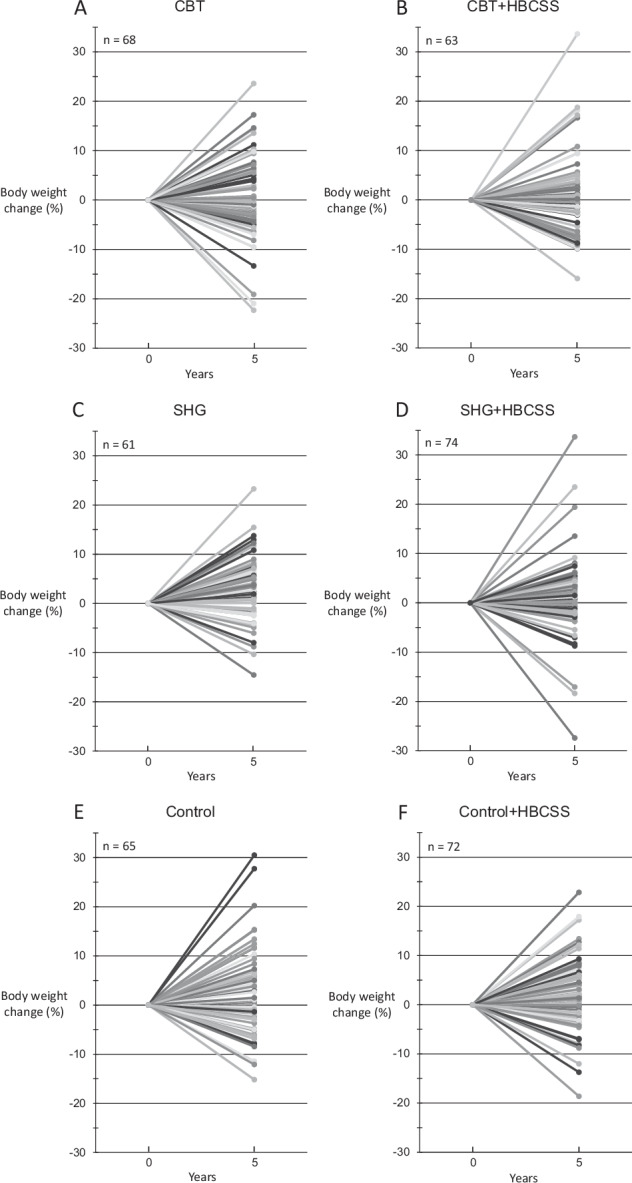
Weight change (%) between baseline and five years among each participant (completers). Panels (**A**, **B**) represent CBT groups, (**C**, **D**) SHG groups and (**E**, **F**) control groups. HBCSS health behaviour change support system, CBT cognitive behavioural therapy-based group counselling, SHG self-help guidance.

Individual changes in weight between baseline and five years (Fig. 3) shows the heterogeneity in the success of the weight change during the follow-up.

Net area in weight (Supplementary Table 2) in the AUC analysis was statistically significantly more negative in groups using the HBCSS compared with groups without HBCSS (Supplementary Fig. 1). The mean weight net area was –3.47 (*p* = 0.018) in the combined HBCSS groups and 1.34 (*p* = 0.340) in non-HBCSS groups, with significant difference between groups (*p* = 0.018) (Supplementary Table 2). CBT + HBCSS group had the most negative net area –9.06 (*p* < 0.001 within group and *p* = 0.035 between the three HBCSS groups). There were no significant differences between the groups without HBCSS.

### Proportions of weight change

**Fig. 4:**
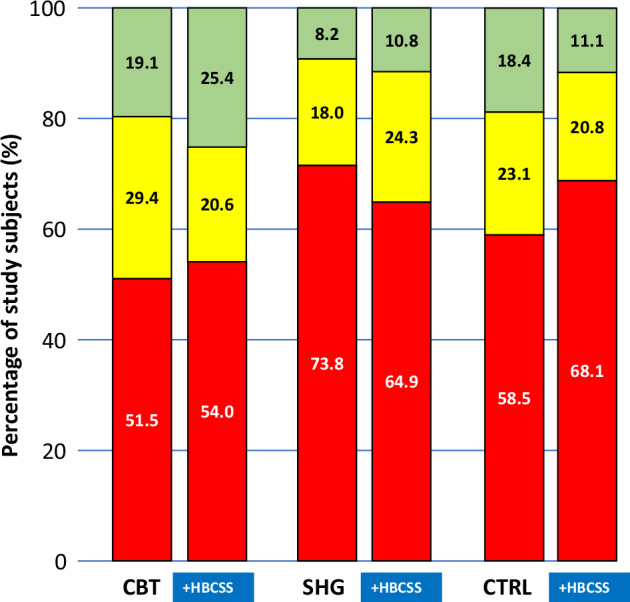
Success of weight loss at five years among completers. Green bar represents weight loss ≥5%, yellow bar 0–4.99% and red bar weight gain. HBCSS health behaviour change support system, CBT cognitive behavioural therapy-based group counselling, SHG self-help guidance, CTRL control.

Dividing the participants into three groups by success in weight loss (weight gain, weight loss 0–4.99% and weight loss ≥5%) demonstrates the proportions of weight change. A weight reduction of 5% or more is considered clinically significant. At five years, the succeeders lost weight regardless of using HBCSS (Fig. 4). Among completers, the clinically significant weight reduction of ≥5% was recorded in the range of 10.8–25.4% of participants in groups using HBCSS and in the range of 8.2–19.1% of participants in groups without HBCSS. There were no differences between groups in proportions of weight change (*p* > 0.05).

### Effects on medications

Fewer blood pressure medications were started over the five-year period in the HBCSS group compared with non-HBCSS group (21 vs. 34, *p* = 0.046). The same trend was observed in diabetes medications, but the difference was not statistically significant (8 vs. 13, *p* = 0.230). There were no statistically significant differences in lipid medications started (*p* = 0.669).

## Discussion

We have previously shown that a 52-week-long web-based HBCSS intervention effectively reduces weight for up to two years [8]. Our current extended follow-up study demonstrates that the non-HBCSS group showed significant weight gain from baseline at five years. This is in accordance with previous observational studies showing an average weight gain of about 400 g per year [15]. Among HBCSS users the weight gain was less extensive although there was no statistical difference between the combined HBCSS group and the combined non-HBCSS group.

The net area under the curve analysis (AUC) showed differences in weight favouring the HBCSS intervention groups during the totality of five-year follow-up, reflecting the previously demonstrated effectiveness of the HBCSS intervention for weight loss at two years. After five years, the best results among the three HBCSS groups in the AUC analysis were achieved in the CBT + HBCSS group. There is evidence that the benefits of weight loss associated with the risk of type 2 diabetes, cardiovascular diseases, and other obesity and overweight related conditions are remarkable at over 5% weight loss [16, 17]. Also, more moderate weight loss (from 2% to 5%) is associated with improved fasting glucose and HbA1c levels [17]. Thus, using the HBCSS intervention combined with CBT-based group counselling may lead to a better state of health because the weight remains lower for a longer time. In addition, our finding of the decrease in the need for antihypertension therapy may indicate that the significant weight loss by HBCSS in the early years, as indicated by AUC analyses, has a health-promoting legacy effect reflected on the better blood pressure control.

Previous studies have shown that weight maintenance is a significant problem in treating overweight and obesity [1]. Short-term lifestyle changes can be very successful [2–4]. The patterns of curves from two to five years among the HBCSS users indicate that additional support may be required for sustained lifestyle change results and to prevent regaining weight. To maintain the results in the long term, e.g., face-to-face, or web-based counselling could be needed if weight is regained. One of the potential options is to repeat the HBCSS intervention a few years after the first intervention period. It should be noted that the weight-regain process is also a result of physiological processes, for example, metabolic adaptation and hormonal changes, in addition to psychological and lifestyle-related factors [18].

Our study searched for an effective, low-cost method for treating overweight and obesity. From a public health perspective, web-based interventions in lifestyle change might be desirable because of the excellent opportunity to treat many individuals at a time at a low cost. Also, in the future, the focus must be on preventing overweight and obesity. Prevention of weight gain and maintaining a healthy weight are considered less challenging, less expensive, and potentially more effective than treatment of obesity [19].

The exceptionally long follow-up period, the original randomized trial design, the number of participants, and the anthropometric measurements performed by study personnel support the reliability, quality, and uniqueness of this study. Considering that only 129 (24.2%) participants dropped out between the baseline and five-year visit, the participants were well committed to the trial. The large number of completers increases the reliability of the results of this study. The practical experience and the previous scientific evidence of the difficulty of maintaining weight loss are also in line with our findings. This study did not include an analysis of the factors influencing the weight gain of the subjects, such as hormonal changes, psychological factors, and life situations.

## Conclusions

Our extended follow-up study shows that the previously demonstrated 2-year weight benefits of the 12-month HBCSS intervention were not maintained at 5 years better than with the non-HBCSS control intervention. However, there were significant weight differences favouring HBCSS over the whole 5-year period, with HBCSS + CBT-based group counselling having the most beneficial effect on weight among the three HBCSS groups. The legacy effect on the need of blood pressure medications supports the benefits of the HBCSS when aiming to lower the risk of cardiovascular diseases. In the future, there is a particular need for an effective tool for maintaining weight loss.

## Supplementary information

Supplementary tables and figures

## Data Availability

The data that support the findings of this study are available on request from the corresponding author. The data are not publicly available due to privacy or ethical restriction.
