# Supplementary material for: Five-year follow-up of a randomized weight loss trial on a digital health behaviour change support system
Source: Int J Obes (Lond). 2025 Mar 15;49(5):949–53. doi: 10.1038/s41366-025-01742-4 (PMC12095081; doi:10.1038/s41366-025-01742-4)
Supplement: Supplementary file 1 — Supplementary tables and figures [file 41366_2025_1742_MOESM1_ESM.docx]

**SUPPLEMENTARY TABLES AND FIGURES**

**Five-year follow-up of a randomized weight loss trial on a digital health behaviour change support system**

Eero Turkkila, Taru Pekkala, Heta Merikallio, Marko Merikukka, Laura Heikkilä, Janne Hukkanen, Harri Oinas-Kukkonen, Tuire Salonurmi, Anna-Maria Teeriniemi, Terhi Jokelainen, Markku J. Savolainen

**Supplementary Table 1*.*** Change in body weight (kg) at five years. Changes are also presented as percentages.

| All subjects | **Body weight change (kg)** | | | **Body weight change (%)** | |
| --- | --- | --- | --- | --- | --- |
| **Group** | **Mean** | **95% CI** | ***p*-value** | **Mean** | **95% CI** |
| CBT (n = 85) | 0.5 | -1.3–2.8 | 1.000 | 0.6 | -1.5–3.0 |
| CBT + HBCSS (n = 88) | 0.6 | -1.8–2.9 | 1.000 | 0.7 | -2.1–3.2 |
| SHG (n = 87) | **2.8** | **0.5–5.2** | **0.010** | **3.1** | **0.6–5.6** |
| SHG + HBCSS (n = 92) | 1.5 | -0.7–3.7 | 0.405 | 1.7 | -0.8–4.0 |
| Control (n = 89) | 1.8 | -0.5–4.1 | 0.242 | 2.0 | -0.6–4.5 |
| Control + HBCSS (n = 91) | 1.7 | -0.5–3.9 | 0.247 | 1.9 | -0.6–4.3 |
| Between groups |  |  | > 0.05 |  |  |
| No HBCSS (n = 261) | **1.7** | **0.3–3.0** | **0.005** | **1.9** | **0.3–3.3** |
| HBCSS (n = 271) | 1.3 | -0.02–2.6 | 0.056 | 1.5 | -0.02–2.9 |
| Between groups |  |  | > 0.05 |  |  |

HBCSS, health behaviour change support system; CBT, cognitive behavioural therapy-based group counselling; SHG, self-help guidance. Linear mixed model was used to examine the differences in weight change (kg).

**A B**

**No HBCSS**

**HBCSS**

0

1

2

5

Years

Digital
intervention

12 months

0

1

2

5

Years

3

2

1

0

-1

-2

-3

-4

-5

3

2

1

0

-1

-2

-3

-4

-5

Body weight
change (%)

Body weight
change (%)

CBT

SHG

Control

**Supplementary Figure 1.** AUC analysis of the body weight change as percentages from baseline at each visit until the five-year visit. Body weight change is presented in figures A and B with or without 12-month (52-week) digital health behaviour change support, respectively. Data is presented in means (%) ± SEM. HBCSS, health behaviour change support system; CBT, cognitive behavioural therapy-based group counselling; SHG, self-help guidance.

**Supplementary Table 2.** Incremental Area Under the Curve analysis of weight change (%) after five years between groups with and without HBCSS.

| **Weight** year*percent (mean net area) (CI 95%) | **HBCSS** | **No HBCSS** | p-value between groups |
| --- | --- | --- | --- |
| Groups combined | **–3.47 (–6.33 to –0.60)**  (n = 242)  ***p* = 0.018** | 1.34 (–1.43 to 4.11)  (n = 226)  *p* = 0.340 | p = 0.018 |
| CBT-counselling (eight sessions) | **–9.06 (–14.54 to –3.59)**  (n = 75)  ***p* < 0.001** | –1.33 (–6.39 to 3.74)  (n = 78)  *p* = 0.603 | *p* = 0.242 |
| SHG-counselling (two sessions) | –0.78 (–5.56 to 4.00)  (n = 82)  *p* = 0.747 | 3.19 (–1.40 to 7.77)  (n = 71)  *p* = 0.170 | *p* = 0.872 |
| Control | –1.12 (–5.80 to 3.56)  (n = 85)  *p* = 0.635 | 2.35 (–2.48 to 7.18)  (n = 77)  *p* = 0.336 | *p* = 0.914 |
|  | *p*-value between groups  ***p* = 0.035** | *p*-value between groups  p = 0.377 |  |

HBCSS, health behaviour change support system; CBT, cognitive behavioural therapy-based group counselling; SHG, self-help guidance.
